# Comprehensive analysis of mitochondrial and nuclear DNA variations in patients affected by hemoglobinopathies: A pilot study

**DOI:** 10.1371/journal.pone.0240632

**Published:** 2020-10-22

**Authors:** Ylenia Barbanera, Francesco Arcioni, Hovirag Lancioni, Roberta La Starza, Irene Cardinali, Caterina Matteucci, Valeria Nofrini, Antonella Roetto, Antonio Piga, Paola Grammatico, Maurizio Caniglia, Cristina Mecucci, Paolo Gorello

**Affiliations:** 1 Department of Medicine, Hematology, University of Perugia, Perugia, Italy; 2 Pediatric Oncohematology, Hospital Santa Maria della Misericordia, Perugia, Italy; 3 Department of Chemistry, Biology and Biotechnology, University of Perugia, Perugia, Italy; 4 Department of Clinical and Biological Sciences, University of Turin, Hospital San Luigi Gonzaga, Turin, Italy; 5 Department of Molecular Medicine, Laboratory of Medical Genetics, San Camillo-Forlanini Hospital, Sapienza University, Rome, Italy; Universita degli Studi di Pavia, ITALY

## Abstract

The hemoglobin disorders are the most common single gene disorders in the world. Previous studies have suggested that they are deeply geographically structured and a variety of genetic determinants influences different clinical phenotypes between patients inheriting identical β-globin gene mutations. In order to get new insights into the heterogeneity of hemoglobin disorders, we investigated the molecular variations on nuclear genes (i.e. *HBB*, *HBG2*, *BCL11A*, *HBS1L* and *MYB)* and mitochondrial DNA control region. This pilot study was carried out on 53 patients belonging to different continents and molecularly classified in 4 subgroup: β-thalassemia (*β*^*+*^*/β*^*+*^, *β*^*0*^*/β*^*0*^ *and β*^*+*^*/β*^*0*^)(15), sickle cell disease *(HbS/HbS*)(20), sickle cell/β-thalassemia (*HbS/β*^*+*^ *or HBS/β*^*0*^)(10), and non-thalassemic compound heterozygous (*HbS/HbC*, *HbO-Arab/HbC*)(8). This comprehensive phylogenetic analysis provided a clear separation between African and European patients either in nuclear or mitochondrial variations. Notably, informing on the phylogeographic structure of affected individuals, this accurate genetic stratification, could help to optimize the diagnostic algorithm for patients with uncertain or unknown origin.

## Author summary

Worldwide most frequent genetic diseases are represented by a very heterogeneous group of congenital hemolytic anaemias which include β-thalassemia and sickle cell anaemia. They are deeply geographically structured and characterized by high complexity of the genetic background associated with variable clinical severities. Due to this heterogeneity we investigated the nuclear and mitochondrial DNA marker variations in patients with hemoglobinopathies in order to provide the first comprehensive genetic stratification of these hereditary disease. This study contributes to detail the multifaceted picture of hemoglobin disorders.

## Introduction

Worldwide, the most frequent genetic diseases are represented by a very heterogeneous subgroup of congenital hemolytic anaemias, which includes β-thalassemia and sickle cell anaemia [1]. Individuals with β-thalassemia have a diverse range of clinical manifestations, from an asymptomatic state to a transfusion-dependent form, depending mainly on the β-globin gene mutations [2,3]. However, individuals with identical β-thalassemia genotypes can exhibit variable clinical severities [4], because of the high complexity of the genetic background associated with the disease [5,6]. The term sickle cell disease (SCD) encompasses a group of disorders characterized by the presence of at least one hemoglobin S allele (HbS; p.Glu6Val in *HBB*) and a second *HBB* allele carrying pathogenic variant resulting in abnormal hemoglobin polymerization. SCD (HbS/HbS) caused by the homozygous *HBB* variant p.Glu6Val is the most common cause of SCD in the US. SCD caused by compound heterozygous *HBB* pathogenic variants includes sickle-hemoglobin C disease (HbS/HbC) and two types of sickle β-thalassemia (HbS/β^+^-thalassemia and HbS/β°-thalassemia). Other β-globin chain variants such as D-Punjab, O-Arab, and E also result in SCD when inherited with HbS. The marked variability in phenotype severity is due to the different genotypes of the disease and the co-presence of other independent genetic factors that may worsen or alleviate the phenotype [7,8]. Single nucleotide polymorphism (SNPs) in several genetic modulators and *cis*-regulatory elements involved in the regulation of human HbF, i.e. *HBG2*, B cell CLL/lymphoma 11A (*BCL11A*), Kruppel-like factor 1 (*KLF1*) and *MYB*, can delay fetal-to-adult hemoglobin (Hb) switch, ameliorating the severity of β-thalassemia and sickle cell disease [9–20].

Other studies focusing on different human populations reported that the SNPs are deeply geographically structured and further loci i.e *KCNK10*, *GPR65*, *RNASE2*, *RNASE3*, and *C/EBPE* are involved in HbF expression regulation [21–25].

In the last decades, mitochondrial DNA (mtDNA) has been proven to be an effective tool to reconstruct the maternal origins of individuals and geographically map samples, but it also plays a key role in various human diseases. Due to its maternal inheritance and the lack of recombination [26,27], its differentiation is generated only by the sequential accumulation of new mutations from a female ancestor that makes the mtDNA a molecular register along maternal lines. Over millennia, the molecular differentiation gave rise to groups of mtDNAs that share a set of variants (called haplotypes) acquired from the same common female ancestor. Even though whole genomic approaches are now answering to questions related to the origin and diversification of species, the mtDNA continues to be an important source of information to reconstruct the genetic history of specific populations. Studies based on sequence variation especially in the noncoding control region have allowed the classification of distinct geographic and ethnic affinities [28–34].

Nowadays, a detailed worldwide mtDNA phylogeny is available, where different haplogroups are defined by specific mutational motifs and restricted to specific areas and population groups [35]. The development of phylogeography is closely related to mtDNA analysis, which was described as one of the best tools to study population variability [36].

The consequences of mitochondrial mutations have been also evaluated in human ageing as well as inherited and acquired diseases [37,38], however the only study focusing on the association of mtDNA variations with β-thalassemia claimed that mitochondrial mutations may increase disease susceptibility or the development of phenotype-associated disease in affected individuals, although concluded that there was no strong proof for the association of these polymorphisms with β-thalassemia [39]. Doubtless, as mentioned above, there should be a correlation between hemoglobinopathies and geographic origins of patients [40,41], that could be deepened also by considering the mtDNA variability.

The aim of this pilot study was to provide a first comprehensive analysis of hemoglobin and uniparental marker variants in 53 patients with hemoglobinopathies coming from different countries, in order to depict the phylogeographic structure of affected individuals.

## Results and discussion

The study was carried out on 53 patients (M/F: 38/15) affected by hemoglobinophaties who referred to our laboratory at the University of Perugia/Hospital Santa Maria della Misericordia, in Umbria a region of Central Italy.

Except for the first patient for whom we do not know the origin, 16 patients (30.2%) were European, 28 (52.8%) African, four (7.6%) Asian and four (7.6%) American. Complete information about geographical characteristics and genotypes were reported in Table 1, while clinical phenotypes were detailed in S1 Text and S1–S4 Tables.

**Table 1:** Geographic information and α/β genotypes of the 53 patients analysed in this study.

	Sample ID	Sex-Age^a^	Ethnic origin		β genotype				α genotype
			Continent	Country	Allele 1		Allele 2		
					Classical nomenclature	*HGVS nomenclature*	Classical nomenclature	*HGVS nomenclature*	
β-Thalassemia (β^+^/β^+^, β^0^/β^0^ and β^+^/β^0^)	1	M	Unknown	Unkonwn	IVS-I-1(G>A)(β^0^)	*HBB*:*c*.*92+1G>A*	IVS-I-1(G>A)(β^0^)	*HBB*:*c*.*92+1G>A*	αα/αα
	2	M-18	Europe	Albania	codon39 (C>T)(β^0^)	*HBB*:*c*.*118C>T*	codon 44(-C)(β^0^)	*HBB*:*c*.*135delC*	αα/αα
	3*	M-3	Africa	Algeria	- 29(A>G)(β^+^)	*HBB*:*c*.*-79A>G*	codon 8(-AA)(β^0^)	*HBB*:*c*.*25_26delAA*	αα/αα
	4*	F-4	Africa	Algeria	- 29(A>G)(β^+^)	*HBB*:*c*.*-79A>G*	codon 8(-AA)(β^0^)	*HBB*:*c*.*25_26delAA*	αα/αα
	5	M	Europe	Italy	codon39 (C>T)(β^0^)	*HBB*:*c*.*118C>T*	IVS-I-6(T>C)(β^++^)	*HBB*:*c*.*92+6T>C*	αα/αα
	6	M-1	Europe	Albania	IVS-I-110(G>A)(β^+^)	*HBB*:*c*.*93-21G>A*	IVS-I-110(G>A)(β^+^)	*HBB*:*c*.*93-21G>A*	αα/αα
	7*	M-15	America	Venezuela	codon17(A>T)(β^0^)	*HBB*:*c*.*52A>T*	- 29(A>G)(β^+^)	*HBB*:*c*.*-79A>G*	αα/αα
	8	M-18	Europe	Albania	IVS-I-5(G>C)/(β^+^)	*HBB*:*c*.*92+5G>C*	IVS-I-6(T>C)(β^++^)	*HBB*:*c*.*92+6T>C*	αα/αα
	9	F-62	Europe	Italy	IVS-I-6(T>C)/(β^++^)	*HBB*:*c*.*92+6T>C*	IVS-I-6(T>C)/(β^++^)	*HBB*:*c*.*92+6T>C*	αα/αα
	10	F-31	Europe	Italy	Codon 39(C>T)(β^0^)	*HBB*:*c*.*118C>T*	IVS-I-6(T>C)/(β^++^)	*HBB*:*c*.*92+6T>C*	αα/αα
	11*	M-3	Asia	Syria	codon 5 (-CT)(β^0^)	*HBB*:*c*.*17_18delCT*	IVS-I-6(T>C)/(β^++^)	*HBB*:*c*.*92+6T>C*	αα/αα
	12*	M-4	Asia	Syria	codon 5 (-CT)(β^0^)	*HBB*:*c*.*17_18delCT*	IVS-I-6(T>C)/(β^++^)	*HBB*:*c*.*92+6T>C*	αα/αα
	13	F-19	Asia	China	- 28(A>G)(β^+^)	*HBB*:*c*.*-78A>G*	cd17(A>T); AAG(Lys)>TAG(Stop codon)(β^0^)	*HBB*:*c*.*52A>T*	αα/αα
	14	M-29	Europe	Italy	IVS-II-745 (C>G) (β^+^)	*HBB*:*c*.*316-106C>G*	Hb Lepore Boston-Washington	*NG_000007*.*3*:*g*.*63632_71046del*	αα/αα
	15	M-10	Asia	Cambodia	Hb E	*HBB*:*c*.*79G>A*	Hb E	*HBB*:*c*.*79G>A*	αα/αα
**Sickle Cell/Thalassemia (HbS/β**^**+**^ **or β**^**0**^**)**	16*	F-18	Europe	Albania	HbS	*HBB*:*c*.*20A>T*	codon 39(C>T)(β^0^)	*HBB*:*c*.*118C>T*	αα/αα
	17*	M-11	Europe	Albania	HbS	*HBB*:*c*.*20A>T*	codon 39(C>T)(β^0^)	*HBB*:*c*.*118C>T*	ααα ^anti3.7^/αα
	18	F-5	Europe	Albania	HbS	*HBB*:*c*.*20A>T*	codon 39(C>T)(β^0^)	*HBB*:*c*.*118C>T*	αα/αα
	19	M-6	Africa	Ivory Coast	HbS	*HBB*:*c*.*20A>T*	-29(A>G)(β^+^)	*HBB*:*c*.*-79A>G*	αα/αα
	20	M-46	Europe	Albania	HbS	*HBB*:*c*.*20A>T*	IVS-I-6(T>C)(β^++^)	*HBB*:*c*.*92+6T>C*	αα/αα
	21*	F-52	America	Venezuela	HbS	*HBB*:*c*.*20A>T*	-29(A>G)(β^+^)	*HBB*:*c*.*-79A>G*	αα/αα
	22	M-51	Europe	Italy (Sicily)	HbS	*HBB*:*c*.*20A>T*	IVS-I-110(G>A)(β^+^)	*HBB*:*c*.*93-21G>A*	αα/αα
	23	M-2	Europe	Albania	HbS	*HBB*:*c*.*20A>T*	IVS-I-110(G>A)(β^+^)	*HBB*:*c*.*93-21G>A*	αα/αα
	24	M-19	Africa	Guinea	HbS	*HBB*:*c*.*20A>T*	-29(A>G)(β^+^)	*HBB*:*c*.*-79A>G*	αα/αα
	25	M-24	Europe	Albania	HbS	*HBB*:*c*.*20A>T*	codon 39(C>T)(β^0^)	*HBB*:*c*.*118C>T*	ααα^anti3.7^/αα
**Sickle cell disease (HbS/HbS)**	26	M-7	Europe	Albania	HbS	*HBB*:*c*.*20A>T*	HbS	*HBB*:*c*.*20A>T*	αα/αα
	27	M-32	America	Dominican Republic	HbS	*HBB*:*c*.*20A>T*	HbS	*HBB*:*c*.*20A>T*	αα/αα
	28	M-4	Africa	Nigeria	HbS	*HBB*:*c*.*20A>T*	HbS	*HBB*:*c*.*20A>T*	(-) α^3.7^/αα
	29	M-1	Africa	Nigeria	HbS	*HBB*:*c*.*20A>T*	HbS	*HBB*:*c*.*20A>T*	αα/αα
	30	M-4	Africa	Nigeria-Ivory Coast	HbS	*HBB*:*c*.*20A>T*	HbS	*HBB*:*c*.*20A>T*	αα/αα
	31	M-2	Africa	Nigeria-Ivory Coast	HbS	*HBB*:*c*.*20A>T*	HbS	*HBB*:*c*.*20A>T*	αα/αα
	32	M-5	Africa	Nigeria	HbS	*HBB*:*c*.*20A>T*	HbS	*HBB*:*c*.*20A>T*	αα/αα
	33	M-2	Africa	Nigeria	HbS	*HBB*:*c*.*20A>T*	HbS	*HBB*:*c*.*20A>T*	(-) α^3.7^/αα
	34	M-1	Africa	Nigeria	HbS	*HBB*:*c*.*20A>T*	HbS	*HBB*:*c*.*20A>T*	αα/αα
	35	M-49	Europe	Italy	HbS	*HBB*:*c*.*20A>T*	HbS	*HBB*:*c*.*20A>T*	(-) α^3.7^/αα
	36	F-5	Africa	Nigeria	HbS	*HBB*:*c*.*20A>T*	HbS	*HBB*:*c*.*20A>T*	αα/αα
	37	F-1	Africa	Nigeria	HbS	*HBB*:*c*.*20A>T*	HbS	*HBB*:*c*.*20A>T*	αα/αα
	38	M-14	Africa	Congo	HbS	*HBB*:*c*.*20A>T*	HbS	*HBB*:*c*.*20A>T*	(-) α^3.7^/αα
	39	M-1	Africa	Benin	HbS	*HBB*:*c*.*20A>T*	HbS	*HBB*:*c*.*20A>T*	αα/αα
	40	M-12	Africa	Senegal	HbS	*HBB*:*c*.*20A>T*	HbS	*HBB*:*c*.*20A>T*	(-) α^3.7^/αα
	41	M-11	America	Venezuela	HbS	*HBB*:*c*.*20A>T*	HbS	*HBB*:*c*.*20A>T*	αα/αα
	42	M-60	Africa	Nigeria	HbS	*HBB*:*c*.*20A>T*	HbS	*HBB*:*c*.*20A>T*	(-) α^3.7^/- α^3.7^
	43	F-1	Africa	Nigeria	HbS	*HBB*:*c*.*20A>T*	HbS	*HBB*:*c*.*20A>T*	αα/αα
	44	M-10 days	Africa	Congo	HbS	*HBB*:*c*.*20A>T*	HbS	*HBB*:*c*.*20A>T*	αα/αα
	45	M-2	Africa	Nigeria	HbS	*HBB*:*c*.*20A>T*	HbS	*HBB*:*c*.*20A>T*	αα/αα
**Compound heterozygotes (HbS/HbC and HbO-Arab/HbC)**	46	M-38	Africa	Ivory Coast	HbS	*HBB*:*c*.*20A>T*	HbC	*HBB*:*c*.*19G>A*	(-) α^3.7^/αα
	47*	M-2	Africa	Ivory Coast	HbS	*HBB*:*c*.*20A>T*	HbC	*HBB*:*c*.*19G>A*	αα/αα
	48	F-1	Africa	Ghana	HbS	*HBB*:*c*.*20A>T*	HbC	*HBB*:*c*.*19G>A*	αα/αα
	49*	F-14	Africa	Ivory Coast	HbS	*HBB*:*c*.*20A>T*	HbC	*HBB*:*c*.*19G>A*	(-) α^3.7^/αα
	50*	F-16	Africa	Ivory Coast	HbS	*HBB*:*c*.*20A>T*	HbC	*HBB*:*c*.*19G>A*	αα/αα
	51	F-24	Africa	Nigeria	HbS	*HBB*:*c*.*20A>T*	HbC	*HBB*:*c*.*19G>A*	αα/αα
	52	F-59	Africa	Morocco	HbO-Arab	*HBB*:*c*.*19G>A*	HbC	*HBB*:*c*.*364G>A*	αα/αα
	53	M-17	Africa	Morocco	HbS	*HBB*:*c*.*20A>T*	HbC	*HBB*:*c*.*19G>A*	αα/αα

^a^Age at time of genetic testing.

*Related patients.

Homozygotes and compound heterozygotes included: 15 β-thalassemia patients, 20 sickle cell disease (HbS/HbS) patients, ten sickle cell/β-thalassemia (HbS/β^+^ or β^0^) patients, eight compound heterozygotes for Hb variants of which seven HbS/HbC and one HbO-Arab/HbC. Ten patients carried α mutations: eight were heterozygous for α ^-3.7^ and two for ααα ^anti 3.7^ (Table 1). Seventeen different mutations of the *HBB* gene were found: 12 generated β-thalassemic defects, two produced Hb thalassemic variants (i.e. Hb-Lepore and HbE) and three were associated with the production of non-thalassemic Hb variants (HbS, HbC and HbO-Arab) (Table 1).

Overall, β-thalassemia patients carried 17 different gene mutations and 12 different genotypes. Seven out of 15 patients (46.6%) were European, two (13.3%) were African, four (26.7%) were Asian and one (6.7%) was American. No precise information was available for one patient (6.7%) (Table 1).

Sickle cell/β-thalassemia patients were characterized by four different β genotypes while α mutations (ααα ^anti 3.7^) were found in two patients from Albania. Seven out of eleven patients were Europeans (63.6%), two (18.2%) Africans and one (9%) American (Table 1).

All sickle cell disease patients carried the same β genotype, while the presence of the -α^3.7^/αα genotype was demonstrated in five patients and the -α^3.7^/-α^3.7^ genotype was highlighted in one patient. Sixteen out of 20 (80%) were African patients, two were Americans (10%) and two were Europeans (10%) (Table 1).

Moreover, seven out of eight patients included in the cohort of compound heterozygotes who carried HbS/HbC genotype (patients 46–51, 53), while the other patient bore HbC/HbO-Arab genotype (patient 52). -α^3.7^/αα genotype was reported in two HbS/HbC cases. All eight patients were African (Table 1).

### Hemoglobin phenotype modulating SNPs: Geographical distribution

The analysis of the five polymorphic loci related to HbF expression (i.e. *HBG2* rs7482144 [C>T], *BCL11A* rs1427407 [G>T], *BCL11A* rs10189857 [A>G], *HBS1L* rs28384513 [A>C], *HBS1L-MYB* rs9399137 [T>C]), showed *BCL11A rs10189857 [A>G]* as the most frequent polymorphic site, since the allele G was detected in 33/53 patients (21 heterozygotes, 12 homozygotes). Conversely, *HBS1L-MYB rs9399137 [T>C]* was the less frequent polymorphic variation, as the allele C was present only in 6/53 patients (one homozygote and five heterozygotes). As referring to the other SNPs: *HBG2* rs7482144 [C>T], *BCL11A rs1427407 [G>T]* and *HBS1L-MYB [A>C]* were detected in pts 9, 19 and 21 respectively, showing an intermediate frequency. No patients carrying *C/EBPE rs45496295 [C>T]* variation were found (Table 2, S5 Table).

**Table 2:** Molecular analysis of phenotype modulating SNPs, mtDNA sequence variation and geographical distribution of the 53 patients analysed in this study.

	Sample ID	Sex-Age^a^	Ethnic origin		*HBG2*	*BCL11A*		*HBS1L-MYB*	*HBS1L-MYB*	*C/EBPE*	mtDNA		
			Continent	Country	rs7482144 [C>T]	rs1427407 [G>T]	rs10189857 [A>G]	rs28384513 [A>C]^b^	rs9399137 [T>C]	rs45496295 [C>T]	Haplotype ID	Haplogroup	Accession Number
**β Thalassemia (β**^**+**^**/β**^**+**^**, β**^**0**^**/β**^**0**^ **and β**^**+**^**/β**^**0**^**)**	1	M	Unkonwn	Unkonwn	CC	GG	**GG**	AA	TT	CC	HT22	R0a	MT176183
	2	M-18	Europe	Albania	CC	GG	AA	A**C**	T**C**	CC	HT38	H7i1	MT176184
	3*	M-3	Africa	Algeria	C**T**	GG	A**G**	AA	TT	CC	HT02	H1q3	MT176185
	4*	F-4	Africa	Algeria	C**T**	GG	A**G**	AA	TT	CC	HT02	H1q3	MT176186
	5	M	Europe	Italy	CC	GG	AA	AA	TT	CC	HT05	HV1	MT176187
	6	M-1	Europe	Albania	CC	GG	A**G**	A**C**	T**C**	CC	HT23	T2c1d+152	MT176188
	7*	M-15	America	Venezuela	C**T**	GG	**GG**	AA	TT	CC	HT28	D1f1	MT176189
	8	M-18	Europe	Albania	CC	GG	A**G**	A**C**	TT	CC	HT14	H2a5a1	MT176190
	9	F-62	Europe	Italy	CC	GG	**GG**	A**C**	TT	CC	HT37	H7h	MT176191
	10	F-31	Europe	Italy	CC	GG	A**G**	A**C**	T**C**	CC	HT42	H7i1	MT176192
	11*	M-3	W. Asia	Syria	CC	G**T**	A**G**	AA	TT	CC	HT17	L2b1	MT176193
	12*	M-4	W. Asia	Syria	CC	GG	**GG**	AA	TT	CC	HT17	L2b1	MT176194
	13	F-19	E. Asia	China	CC	GG	**GG**	A**C**	TT	CC	HT12	N9a10a	MT176195
	14	M-29	Europe	Italy	CC	GG	AA	AA	TT	CC	HT33	H33c	MT176196
	15	M-10	E. Asia	Cambodia	**TT**	GG	**GG**	**CC**	T**C**	CC	HT16	D4b2d	MT176197
**Sickle Cell/Thalassemia (HbS/β**^**+**^ **or β**^**0**^**)**	16*	F-18	Europe	Albania	CC	GG	**GG**	A**C**	TT	CC	HT15	H76a	MT176198
	17*	M-11	Europe	Albania	CC	GG	**GG**	AA	TT	CC	HT30	X2c1b	MT176199
	18	F-5	Europe	Albania	CC	GG	A**G**	A**C**	**CC**	CC	HT07	J1c2e	MT176200
	19	M-6	Africa	Ivory Coast	CC	GG	A**G**	A**C**	TT	CC	HT04	L2a1a1	MT176201
	20	M-46	Europe	Albania	CC	GG	A**G**	A**C**	TT	CC	HT24	T2b	MT176202
	21*	F-52	America	Venezuela	C**T**	G**T**	A**G**	AA	TT	CC	HT28	D1f1	MT176203
	22	M-51	Europe	Italy (Sicily)	CC	G**T**	AA	AA	TT	CC	HT01	H2a2a1	MT176204
	23	M-2	Europe	Albania	CC	GG	A**G**	AA	TT	CC	HT43	H12a	MT176205
	24	M-19	Africa	Guinea	**TT**	G**T**	AA	AA	TT	CC	HT36	L3f1b+16292+150	MT176206
	25	M-24	Europe	Albania	CC	G**T**	AA	A**C**	T**C**	CC	HT06	J1c+16261	MT176207
**Sickle cell disease (HbS/HbS)**	26	M-7	Europe	Albania	CC	G**T**	A**G**	AA	TT	CC	HT20	T1a1+@152	MT176208
	27	M-32	America	Dominican Republic	CC	GG	**GG**	AA	TT	CC	HT29	B4	MT176209
	28	M-4	Africa	Nigeria	CC	G**T**	AA	AA	TT	CC	HT40	G1a	MT176210
	29	M-1	Africa	Nigeria	CC	GG	AA	AA	TT	CC	HT40	G1a	MT176211
	30	M-4	Africa	Nigeria-Ivory Coast	CC	G**T**	AA	AA	TT	CC	HT32	L2a1	MT176212
	31	M-2	Africa	Nigeria-Ivory Coast	CC	GG	A**G**	AA	TT	CC	HT32	L2a1	MT176213
	32	M-5	Africa	Nigeria	CC	G**T**	AA	AA	TT	CC	HT09	L1b2a	MT176214
	33	M-2	Africa	Nigeria	CC	G**T**	AA	AA	TT	CC	HT21	L1b1a+189	MT176215
	34	M-1	Africa	Nigeria	CC	G**T**	A**G**	A**C**	TT	CC	HT41	L2a1+143	MT176216
	35	M-49	Europe	Italy	CC	G**T**	AA	AA	TT	CC	HT39	L3e1b2	MT176217
	36	F-5	Africa	Nigeria	CC	GG	A**G**	A**C**	TT	CC	HT10	L2a1	MT176218
	37	F-1	Africa	Nigeria	CC	**TT**	AA	A**C**	TT	CC	HT03	L1c1d	MT176219
	38	M-14	Africa	Congo	CC	GG	AA	A**C**	TT	CC	HT08	L1c2a1a	MT176220
	39	M-1	Africa	Benin	C**T**	GG	AA	AA	TT	CC	HT04	L2a1a1	MT176221
	40	M-12	Africa	Senegal	C**T**	GG	AA	AA	TT	CC	HT35	M30c	MT176222
	41	M-11	America	Venezuela	C**T**	GG	**GG**	AA	TT	CC	HT44	U7a	MT176223
	42	M-60	Africa	Nigeria	CC	GG	**GG**	A**C**	TT	CC	HT34	L2a1+16189+(16192)	MT176224
	43	F-1	Africa	Nigeria	CC	GG	AA	A**C**	TT	CC	HT13	L3k1	MT176225
	44	M-10 days	Africa	Congo	CC	G**T**	A**G**	A**C**	TT	CC	HT26	L0a1a2	MT176226
	45	M-2	Africa	Nigeria	CC	GG	AA	AA	TT	CC	HT25	L0a1a	MT176227
**Compound heterozygotes (HbS/HbC and HbO-Arab/HbC)**	46	M-38	Africa	Ivory Coast	CC	G**T**	AA	AA	TT	CC	HT11	L2a1b+143	MT176228
	47*	M-2	Africa	Ivory Coast	CC	G**T**	A**G**	AA	TT	CC	HT31	L2a1+143	MT176229
	48	F-1	Africa	Ghana	CC	GG	**GG**	A**C**	TT	CC	HT18	L3b	MT176230
	49*	F-14	Africa	Ivory Coast	CC	G**T**	A**G**	AA	TT	CC	HT31	L2a1+143	MT176231
	50*	F-16	Africa	Ivory Coast	CC	**TT**	AA	AA	TT	CC	HT31	L2a1+143	MT176232
	51	F-24	Africa	Nigeria	CC	G**T**	A**G**	AA	TT	CC	HT19	L3d1b3a	MT176233
	52	F-59	Africa	Morocco	CC	GG	A**G**	A**C**	TT	CC	HT02	H1q3	MT176234
	53	M-17	Africa	Morocco	CC	GG	A**G**	AA	TT	CC	HT27	M1a2a	MT176235

^a^Age at time of genetic testing.

^b^Only SNPs that reduce HbF.

*Related patients.

**Fig 1:**
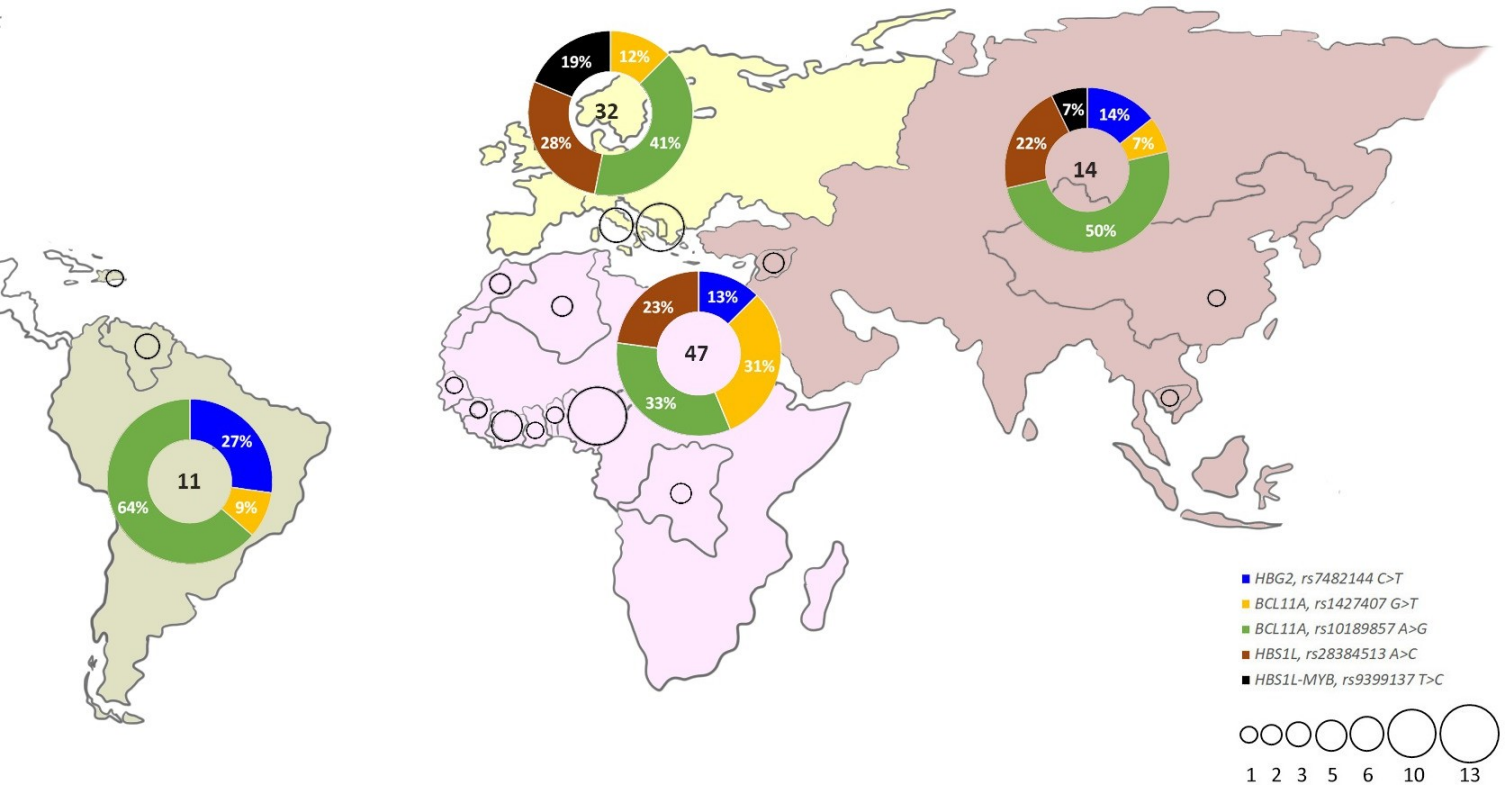
Nuclear SNP frequencies and geographic distribution of five nuclear SNPs in the 53 patients. Black rings represent the number of patients in different geographic areas. Pie charts represent the frequencies of each SNP. The central number indicates the total alleles carrying SNPs.

Interestingly, we observed that frequencies of the five analysed SNPs were related to the geographical origin of patients (Fig 1). Although in our cohort there were only four Asian cases, all investigated SNPs were detected. Conversely, in the other patients (i.e. Europeans, Africans, Americans), the frequency of each SNP was considerably different. *HBS1L-MYB* rs9399137 T>C was not detected in both our African or American populations, while *HBG2* rs7482144 C>T was not detected in European patients (Fig 1, Table 2, S1A and S1B Fig).

In particular, *HBG2* rs7482144 [C>T] was found in nine patients, five African, three Americans and one Asian (S1A Fig, Table 2). The absence of this variation in European subjects did not completely fit with the allele frequencies retrieved from GnomAD database, which reported T alleles in the European population, although in a lower frequency (3839 T alleles and 10893 C alleles) (S1B and S1C Fig).

*BCL11A* rs1427407 [G>T] spread over all analysed ethnic group: 13 African patients, four Europeans, one American and one Asian. Likewise, *BCL11A*, rs10189857 [A>G] was present in 33/53 patients: 14 Africans, ten Europeans, four Asians, four Americans and no precise ethnic information were available for one patient (S1A Fig, Table 2). The allele frequencies of both *BCL11A* rs1427407 and *BCL11A* rs10189857 were in agreement with those reported in GnomAD database (Fig 1B and 1C).

*HBS1L-MYB* rs28384513 [A>C] was identified in ten African patients, nine Europeans and two Asians, while it was not detected in American patients (Fig 1A, Table 2). Notably GnomAD database reported the G allele also in this population, although in a lower frequency (9164 T alleles and 5824 G alleles) (S1B and S1C Fig). The low number of American patients included in our cohort (i.e. three pts) could explain the discrepancy between our results and the database.

Finally, the *HBS1L-MYB* rs9399137 [T>C], was detected only in 6/53 analysed patients, five European patients and one Asian (S1A and S1B Fig, Table 2). No African patients with *HBS1L-MYB* rs9399137 [T>C] were found in our cohort although this represented the largest group. Allele frequencies obtained by GnomAD database reported C alleles in American and African populations, although at lower frequencies (S1B and S1C Fig).

In conclusion, for three out of five SNPs our results were in agreement with GnomAD database. The only discrepancy regards *HBG2 rs7482144 [C>T]* which was not detected in European patients and *HBS1L-MYB* rs9399137 [T>C] which was not detected in Africans although these patients represented 30.2% and 52.8% of the cohort, respectively. These discrepancies may be due to a selection bias as European patients represented only Italian and Albania geographical areas, while 24/28 African patients came from seven Sub-Saharan countries and only 4/28 came from two North Africa countries. (Fig 1)

### Analysis of mtDNA sequence variation and integration with hemoglobin marker variants

Mitochondrial control region sequences ranged from nucleotide position (np) 16024 to np 210, thus encompassing the HVSI and most of the HVSII (Table 2). The overall sequence alignment of samples revealed 99 polymorphic sites (S), represented by ten transversions and two indel (one deletion and one insertion). Nucleotide diversity (π) across all samples was estimated at 0.0173. The haplotype diversity was very high (Hd = 0.993) as well as the average number of nucleotide differences (k = 13,052).

**Fig 2:**
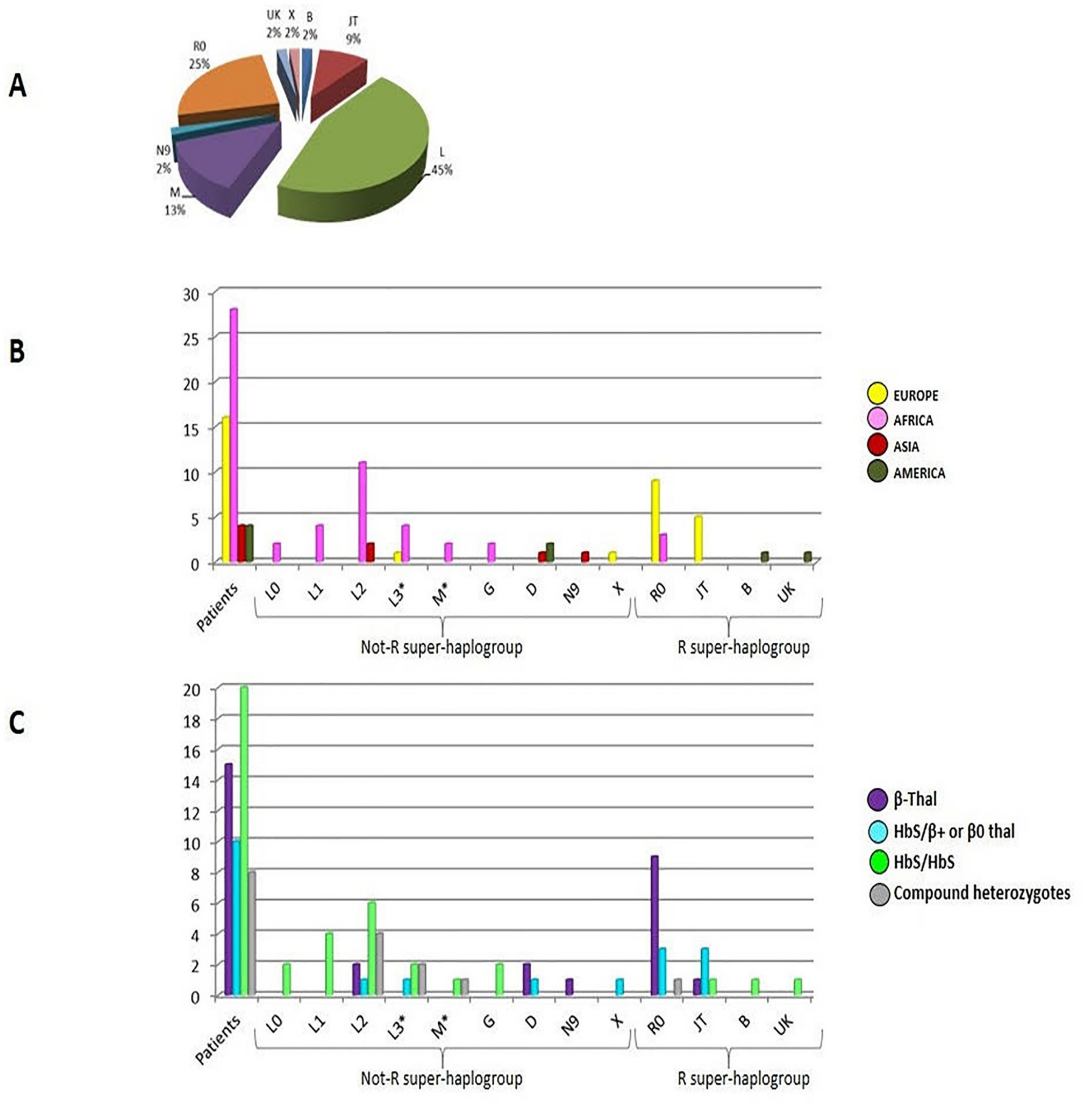
Haplogroup frequency. (A) Haplogroup frequency pie chart. (B) Geographic distribution of patients based on their declared origin and reported for different haplogroups. (C) Distribution of hemoglobinopathies subgroups in all patients for different mtDNA haplogroups.

When aligned to the revised Cambridge Reference Sequence, a total of 44 different haplotypes were obtained and classified through their mutational motifs into 13 different macro-haplogroups: B, D, G, JT, L0, L1, L2, L3*, M*, N9, R0, UK and X (Fig 2A). Seven haplotypes were shared between two or three samples, but five couples of those individuals declared to be maternally related. For the remaining two haplotypes, one (HT02) was found in two brothers from Algeria and one sample from Morocco the other (HT04) was detected in one patient from Ivory Coast and one from Benin.

**Fig 3:**
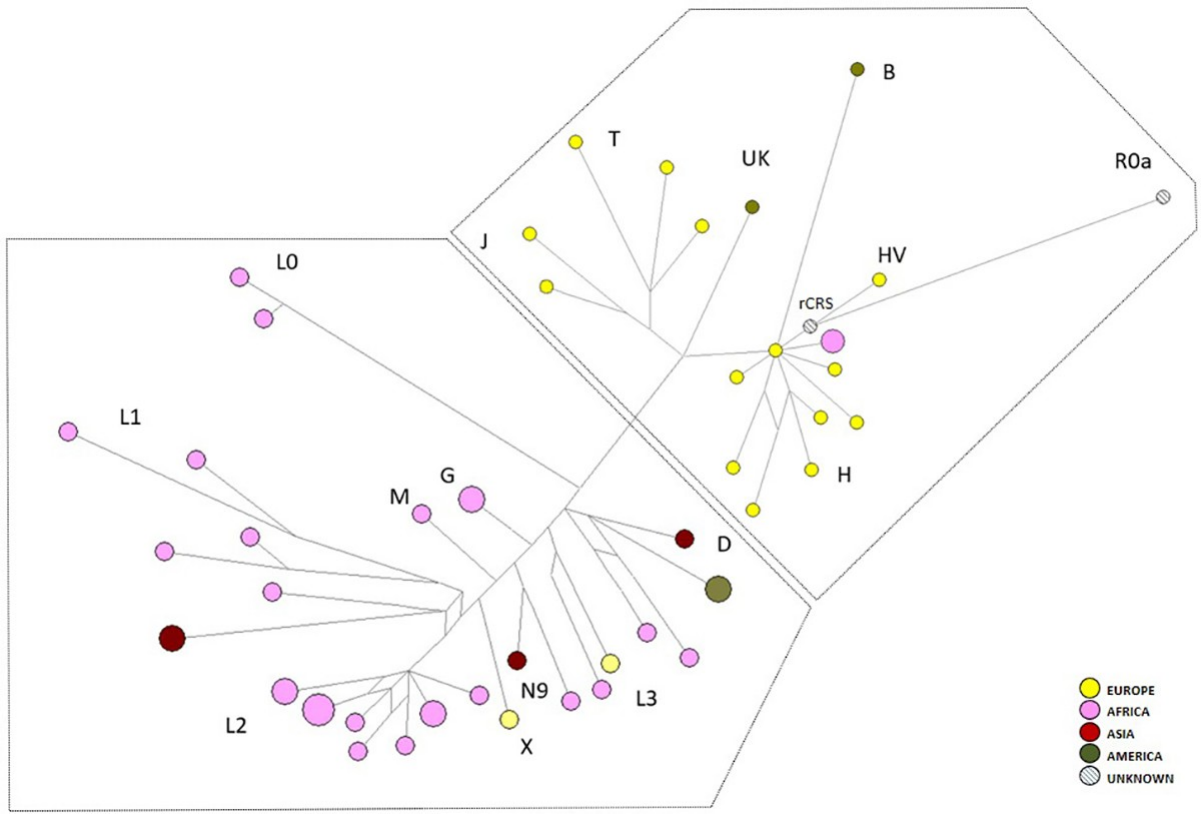
Network of mitochondrial variation. Tree of the 53 mtDNA haplotypes observed among the patients, highlighted with different colors according to their geographic origin. The circles are proportional to the number of samples carrying the same haplotype. Haplogroups and sub-haplogroups are indicated with capital letters. Super-haplogroups R and not-R are defined in the right and left box, respectively.

In order to better define the phylogenetic relationships between samples, we have constructed the network illustrated in Fig 3. Haplogroup labels are reported according to the Phylotree nomenclature [42]. The network topology showed two major mtDNA clusters, corresponding to super-haplogroups R and not-R. Within R, JT and H haplogroups prevailed. The latter formed a star-like cluster mainly represented by sub-haplogroups H1, H2 and H7. R0a, HV, B and UK were also represented. The not-R cluster included mostly L1, L2 and L3* sub-lineages, followed by L0 and D; also N9, M, X and G were revealed.

Among samples, the most represented lineage belonged to the super-haplogroup L (45%), consisting in L0 (8%), L1 (17%), L2 (54%) and L3* (21%), followed by R0 (25%) and M* (13%) (Fig 2B). The sibling haplogroup JT was detected with 9% of frequency, while B, N9, UK and X lineages were represented by only one sample each.

It is known that macro-haplogroup L represents the most ancestral mitochondrial lineage of all living modern humans and, if excluding the derived L3 branches M and N, it represents the majority of the typical sub-Saharan mtDNA variability. Within L, L2 is the most common haplogroup in Africa and it has been previously observed throughout the continent [43]. Our findings agree with the haplogroup frequency and origin of our samples specifically from Nigeria and Ivory Coast (but also Congo and Benin), except for Syria, from which two samples (brothers) derived. L3* was found in two samples from Nigeria, one from Guinea, one from Ghana and one from Italy. Even if L haplotypes account for <1% of mtDNAs in Europe, with L1b being the most common haplogroup, frequencies widely change, ranging from 3% in Southern Europe to 0.7% in Central Europe and 0.5% in Northern Europe.

Within R0, only one sample R0a and one HV1 were found, while the remaining patients were H, thus corresponding to a total of 11/53 (21%). Haplogroup H is the most frequent mitochondrial lineage in Europe (~40%) with a declining pattern from Western Europe towards the Near East and Caucasus (~10–20%). It was also found in Africa, especially the North region, with a peak of H1 in Maghreb and this is in accordance with the origin of our H samples (Italy, Albania, Morocco and Algeria).

As for the macro-haplogroup M, samples originated from Venezuela and Cambodia and belonged to the D4 sub-branches (D1f1 and D4b2d), which exactly reflect the geographic maximum distribution of these lineages (South America and East Asia, respectively) [44]. Even patient 53 from Morocco, belonging to M1a2a mitochondrial haplogroup, was congruent with the frequency distribution of the lineage, unlike African patients 28 and 29 (brothers) and 40, which belonged to G1a and M30c respectively, both sub-lineages typical of Asia.

The East Asian B4 haplogroup revealed in patient 27 from Dominican Republic could result odd, but we should keep in mind that a subclade of B4b is one of the five haplogroups found among indigenous people of the Americas, thus explaining a lower distribution also in Central America.

Patient 13 from China belonging to N9a10a perfectly reflects the East Asian origin and the distribution peak of the lineage [45].

After evaluating relationships between mitochondrial phylogeny and the geographic origin of individuals, we moved to a different level of resolution, including also the nuclear SNP data in a new network (S2 Fig, Table 2). We considered the mitochondrial control-region haplotypes, whose phylogenetic relationships are illustrated in the Fig 3, together with zygosity of the five variable genes, trying to detail the patients’ genotype. Patients 5, 14, 29 and 45 were the only homozygous wildtype for all variables: two from Italy (both belonging to the R0 haplogroup) and two from Nigeria (carrying L0 and G mitogenomes). A total of 41/53 patients showed the prevalence of wildtype homozygosity, while only six individuals (patients 6, 10, 21, 25, 34 and 44) were heterozygous for three polymorphisms. Fourteen patients were homozygous mutants for one SNP, while only patient 15 was homozygous mutant for three variables. It is to be highlighted that all individuals within L mitochondrial macro-haplogroup were homozygous wildtype for *HBS1L-MYB* rs9399137 [T>C], as patients harbouring the JT mitochondrial lineage were homozygous wildtype for *HBG2* rs7482144 [C>T]. Conversely, *C/EBPE* rs45496295 [C>T] was always found as homozygous wildtype, thus it was excluded from the representation.

A further analysis was performed, trying to obtain a graphical overview of the possible integration between mtDNA control region variability and nuclear mutations associated to hemoglobinopathies subgroups (Table 2, S2 Text and S5 Table). In S3 Fig, different phenotypical subgroups were highlighted in the mitochondrial phylogenetic tree: β-thalassemia (β-thal) patients, sickle cell/β-thalassemia (HbS/β^+^ or β^0^), sickle cell disease (HbS/HbS) and compound heterozygotes (HbS/HbC and HbO-Arab/HbC). The overall network revealed a predominant distribution of the hemoglobinopathies subgroups within clades (S3 Fig). In particular, except for three haplotypes belonging to samples from Albania (patient 26) and South America (patiens 27 and 41) that lied in the R clade, sickle cell disease seemed to be common among not-R haplogroups, especially African lineages (L0, L1, L2 and L3*). It is to be noticed that no sickle cell disease patients belonged to macro-haplogroup R0 (inclusive of R0a, H and HV haplogroups) (Fig 2C). A similar distribution was observed for compound heterozygotes patients, all Africans; they lied in not-R clade (M1, L2 and L3* haplogroups), except for patient 52, from Morocco, that belonged to H1 lineage and it was the only patient characterized by HbC/HbO-Arab genotype. An opposite distribution could be observed for thalassemic patients, which spread among R haplogroups, except for four samples from South America (patient 7), Syria (patients 11 and 12, brothers) and Cambodia (patient 15). In particular, the R0 clade seemed to be overall characterized by β-thalassemic individuals. On the other hand, sickle cell/β-thalassemia patients (HbS/β^+^ or β^0^) seemed to be widespread in the network.

When we separated data in four different networks, one for each hemoglobinopathies subgroup coloured with the geographic origin of the patient, it was clear that Asian samples were all thalassemic, but clustered far from European thalassemic patients (S3E Fig). No African patient was affected by thalassemia (with the exception of a couple of brothers from Algeria). If excluding two samples with sickle cell/β-thalassemia patients (HbS/β^+^ or β^0^) (S3F Fig) Africans prevail in sickle cell disease grouping (S3G Fig) and are the only source of compound heterozygotes (S3H Fig). No star-like pattern of molecular relatedness emerged that would represent any event of local expansion.

To resume, we observed that European patients fall prevalently into the thalassemic (β^+^/β^+^, β^0^/β^0^ and β^+^/β^0^) or sickle cell/β-thalassemia (HbS/β^+^ or β^0^) subgroups, while Africans into the remaining two (i.e. HbS/HbS and HbS/HbC) (S4 Fig).

**Fig 4:**
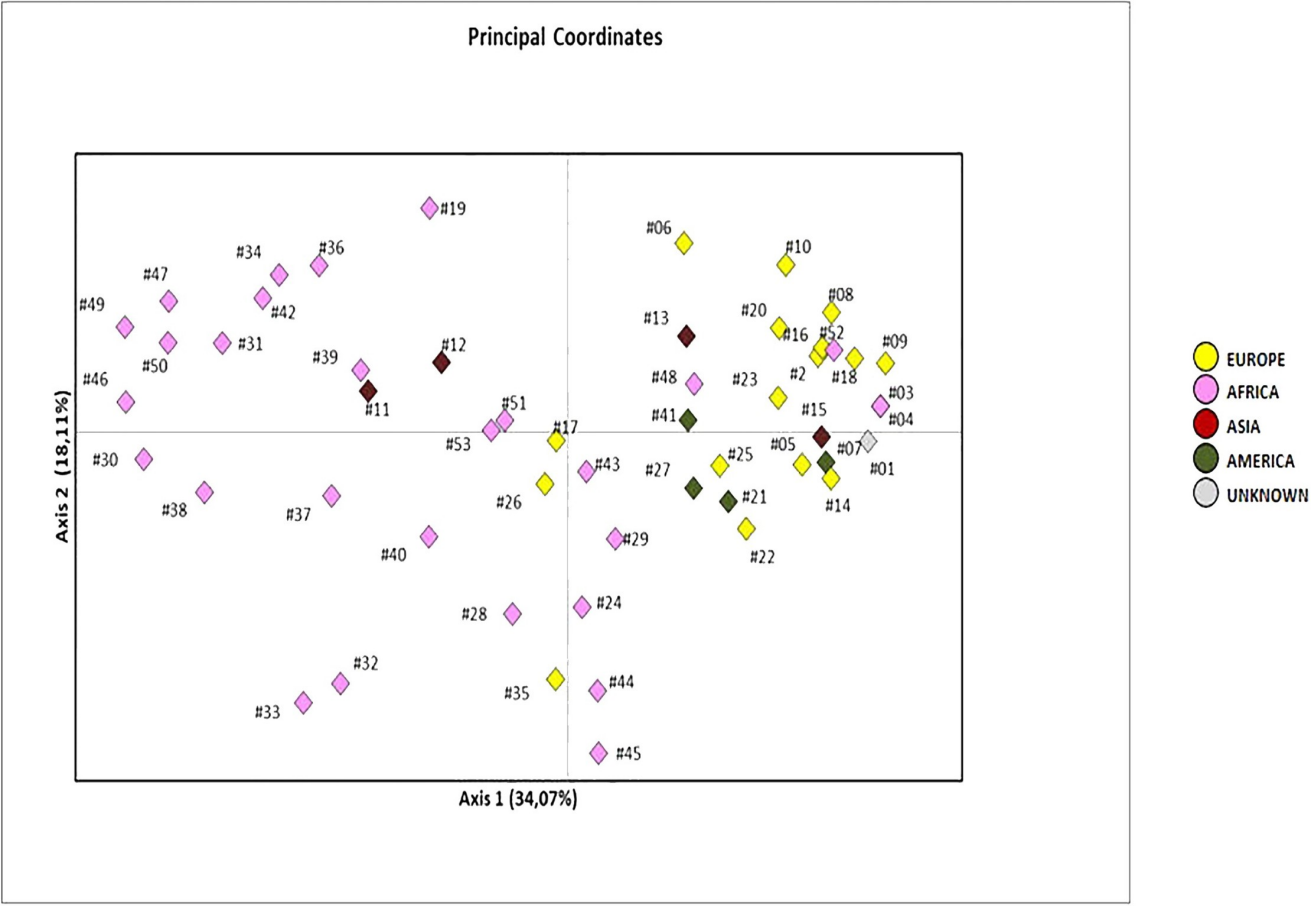
Two-dimensional Principal Component Analysis plot obtained by including all mitochondrial and nuclear DNA variations, considered separately in each sample and coloured depending on their geographic origin.

The clear structuring and sub-division between African and European clades could be detected also in the principal component analysis (PCA) performed by considering all variable sites (both nuclear and mtDNA SNPs) for the 53 patients (Fig 4). Moreover, American samples lied in two quarters, close to Europeans. The graph showed also three European samples close to the African quarters (left quarters): Albanian patients 17 (X haplogroup) and 26 (JT), and patient 35 (L3*) from Italy.

On the other hand, five African samples stand close to the vertical line that separates left and right quarters: patient 24 (L3*) from Guinea; patients 29 (G), 43 (L3*), and 45 (L0) from Nigeria; and patient 44 (L0) from Congo. Interestingly, all these patients together with three Europeans (patients 17, 26, 35) carried at least HbS allele, which is very frequent in the Sub Saharan Africa. The prevalently European quarter (the first quarter on the right) shows four African patients (patients 3 and 4 siblings, and patients 48 and 52). The two most distant patients from African samples are from North Africa (Morocco and Algeria) confirming the closeness to European ones. Intriguingly, only two thalassemic samples lie on the left half of the PCA: they are patients 11 and 12, the only samples from West Asia, while the other two Asian samples were from China and Cambodia, and fall into the right half of the graph.

Even if the past and recent migrations could partially alter the picture, a high level of population and geographic structuring still persist.

## Conclusions

This study represents the first comprehensive analysis of nuclear and mitochondrial DNA marker variations in patients with hemoglobinopathies and showed a clear separation between African and European patients allowing for depicting the phylogeographic structure of affected individuals. Further investigations on a larger cohort of patients, as well as on additional mitochondrial and nuclear variants using standard and Next Generation Sequencing methodologies, will allow to establish possible correlations between genetic data and clinical behaviour. Taking all together, this pilot study based on hemoglobin and uniparental marker variants could represent an integrated and powerful tool for enliven the multifaced pictures of hemoglobinophaties.

## Materials and methods

### Patients and DNA extraction

The study was carried out on 53 patients (M/F: 38/15) affected by hemoglobinophaties who referred to our laboratory at the University of Perugia/Hospital Santa Maria della Misericordia, in Umbria a region of Central Italy. (Table 1). α and β genotype of patients 1–12, 16–23, 26–39 and 46–52 were previously described [46]. Moreover patients 3–4, 11–12, 16–17 and 47, 49–50 were brothers. Patient 21 was mother of patient 7.

Peripheral blood samples were collected during routine medical examinations and genomic DNA was isolated through salting-out procedure [47].

At diagnosis and/or during follow-up all patients or their parents/guardians provided informed consent for sample collection, storage and for research collaborative studies. Protocols were approved by the Ethics Committee for Clinical Experimentation of the University of Perugia (protocol no. 2017–01) and analyses were performed in agreement with the Helsinki declaration.

### Genomic DNA analysis

Five SNP associated with phenotype severity modulating respectively *HBG2* rs7482144 [C>T], *BCL11A* rs1427407 [G>T], *BCL11A* rs10189857 [A>G], *HBS1L* rs28384513 [A>C] and *HBS1L-MYB* rs9399137 [T>C] were studied in all patients, using polymerase chain reaction (PCR)-based reverse dot-blot technique (β-Thal Modifier StripAssay; ViennaLab Diagnostics GmbH, Vienna, Austria).

*C/EBPE*, rs45496295 [C>T] were studied using primers: *CEBP_F*: 5’-GACAGGAGCTGGCTCTGAGT-3’ and *CEBP_R*: 5’-TCCGCAGAGTTAGGCCGT-3’

Nucleotide Sequencing was performed using BigDye^TM^ Terminator Cycler Sequencing Kit and ABI PRISM^TM^ 310 Genetic Analyzer (PE Applied Biosystems, Foster City, CA, USA) [48].

The SNP frequency in the different populations were obtained by GnomAD database (Genome Aggregation Database, https://gnomad.broadinstitute.org/).

### Mitochondrial DNA analysis

Mitochondrial control region between sites 15877 and 259 was amplified using two primers designed on the revised Cambridge Reference Sequence (rCRS) with accession number NC_012920 [49]: forward 5’-CAAATGGGCCTGTCCTTGTA-3’ and reverse 5’-TGTGCAGACATTCAATTGTT-3’. The PCR fragments were first enzymatically purified (ExoSAP-IT®, USB Corporation, Cleveland, OH, USA), then cycle sequenced by application of ABI Prism™ BigDye Terminator chemistry on the ABI PRISM^TM^ 310 Genetic Analyzer (PE Applied Biosystems, Foster City, CA, USA), with the forward primer 5’-CCATTAGCACCCAAAGCTA-3’.

Sequences were assembled and aligned to rCRS using Sequencher™ 5.10 (Gene Codes Corporation). Whenever electropherograms showed ambiguities, new PCR amplifications and sequencing reactions were performed.

Final assembling encompassed at least the entire Hyper Variable Sequence HVSI and part of HVSII (from np 16024 to np 210). All mitochondrial control-region sequences were deposited in GenBank with accession numbers MT176183-MT176235.

For each sample, haplotypes were registered and classified in haplogroups according to the respective mutational motifs by referring to HaploGrep2 software based on PhyloTree database build 17, www.phylotree.org [42].

Genetic variation indices were estimated by using DnaSP 5.1 software [50].

The evolutionary relationships among haplotypes were visualized through the construction of a median-joining network using Network 10 software (www.fluxus-engineering.com).

By default, all polymorphisms relative to rCRS were weighted by a value of ten, except for the highly variable site 16519 and the variations in the poly-C stretch around np 16189 that were down-weighted to one. Conversely, triple weights were assigned to the mutational motifs 16223 and 73 in order to strengthen the topology of the network.

Principal component analyses (PCA) were performed using Excel software implemented by XLSTAT, as described elsewhere [51] in order to explain the variance of multivariate data (mitochondrial and nuclear DNA variation, considered separately in each sample).

## Supporting information

S1 Fig Nuclear SNP frequencies. (A) Frequency per SNP of patients reported according to the geographic origin. (B) Frequency per continent of the ancestral allele and the variant one in the cohort of this study. (C) Frequency per continent of the ancestral allele and the variant one reported by GnomAD database. Please note that panels A and B are referred to coding DNA reference sequence while panel C is referred to the genomic reference sequence.
(DOCX)

S2 Fig Network of mitochondrial and nuclear variation. Tree of the 53 mtDNA haplotypes observed among the patients in which zygosity of SNP was manually highlighted (rs45496295 was excluded because samples were all homozygous for it).
(DOCX)

S3 Fig Nuclear SNP frequency, geographic distribution and network mtDNA, in the subgroups of patients. (A) Nuclear SNP frequency and Geographic distribution in β-thalassemia (β^+^/β^+^, β^0^/β^0^ and β^+^/β^0^) patients, (B) sickle cell/β-thalassemia (HbS/β^+^ or β^0^) patients, (C) sickle cell disease patients (HbS/HbS) (D) compound heterozygotes (*HbS/HbC* and HbO-Arab/HbC). Panel E-H: network plots obtained by including all mitochondrial and nuclear DNA variation, considered separately in each pathological group, and colored depending on their geographic origin.
(DOCX)

S4 Fig Network of mitochondrial variation and hemoglobinopathies. Tree of the 53 mtDNA haplotypes observed among the patients in which different subgroups of hemoglobinopathies were highlighted.
(DOCX)

S1 Table Clinical data of β thalassemia patients. (DOCX)

S2 Table Clinical data of sickle cell/thalassemia patients. (DOCX)

S3 Table Clinical data of sickle cell disease patients. (DOCX)

S4 Table Clinical data of compound heterozygotes patient. (DOCX)

S5 Table Molecular characteristics of the genetic modulators of clinical phenotypes in homozygotes and compound heterozygotes for β gene defect. (DOCX)

S1 Text (DOCX)

S2 Text (DOCX)
