# Supplementary material for: Comprehensive analysis of mitochondrial and nuclear DNA variations in patients affected by hemoglobinopathies: A pilot study
Source: PLoS One. 2020 Oct 22;15(10):e0240632. doi: 10.1371/journal.pone.0240632 (PMC7581000; doi:10.1371/journal.pone.0240632)
Supplement: S1 Fig — (A) Frequency per SNP of patients reported according to the geographic origin. (B) Frequency per continent of the ancestral allele and the variant one in the cohort of this study. (C) Frequency per continent of the ancestral allele and the variant one reported by GnomAD database. Please note that panels A and B are referred to coding DNA reference sequence while panel C is referred to the genomic reference sequence. (DOCX) [file pone.0240632.s001.docx]

**S1 Fig.** **Nuclear SNP frequencies.** (A) Frequency per SNP of patients reported according to the geographic origin. (B) Frequency per continent of the ancestral allele and the variant one in the cohort of this study. (C) Frequency per continent of the ancestral allele and the variant one reported by GnomAD database. Please note that panels A and B are referred to coding DNA reference sequence while panel C is referred to the genomic reference sequence.

**
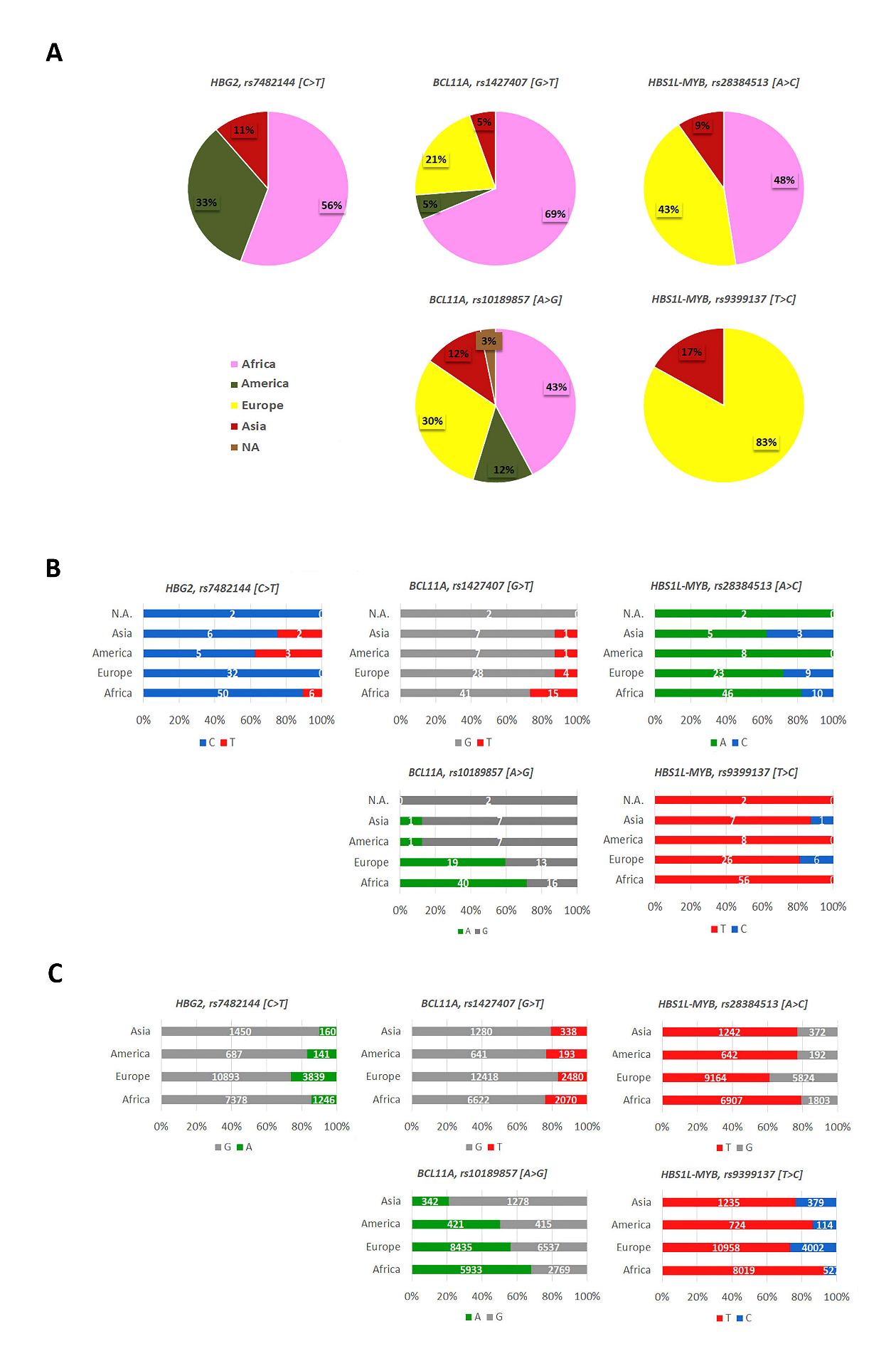
**
