# Supplementary material for: Comprehensive analysis of mitochondrial and nuclear DNA variations in patients affected by hemoglobinopathies: A pilot study
Source: PLoS One. 2020 Oct 22;15(10):e0240632. doi: 10.1371/journal.pone.0240632 (PMC7581000; doi:10.1371/journal.pone.0240632)
Supplement: S2 Fig — Tree of the 53 mtDNA haplotypes observed among the patients in which zygosity of SNP was manually highlighted (rs45496295 was excluded because samples were all homozygous for it). (DOCX) [file pone.0240632.s002.docx]

**S2 Fig. Network of mitochondrial and nuclear variation.** Tree of the 53 mtDNA haplotypes observed among the patients in which zygosity of SNP was manually highlighted (rs45496295 was excluded because samples were all homozygous for it).

**
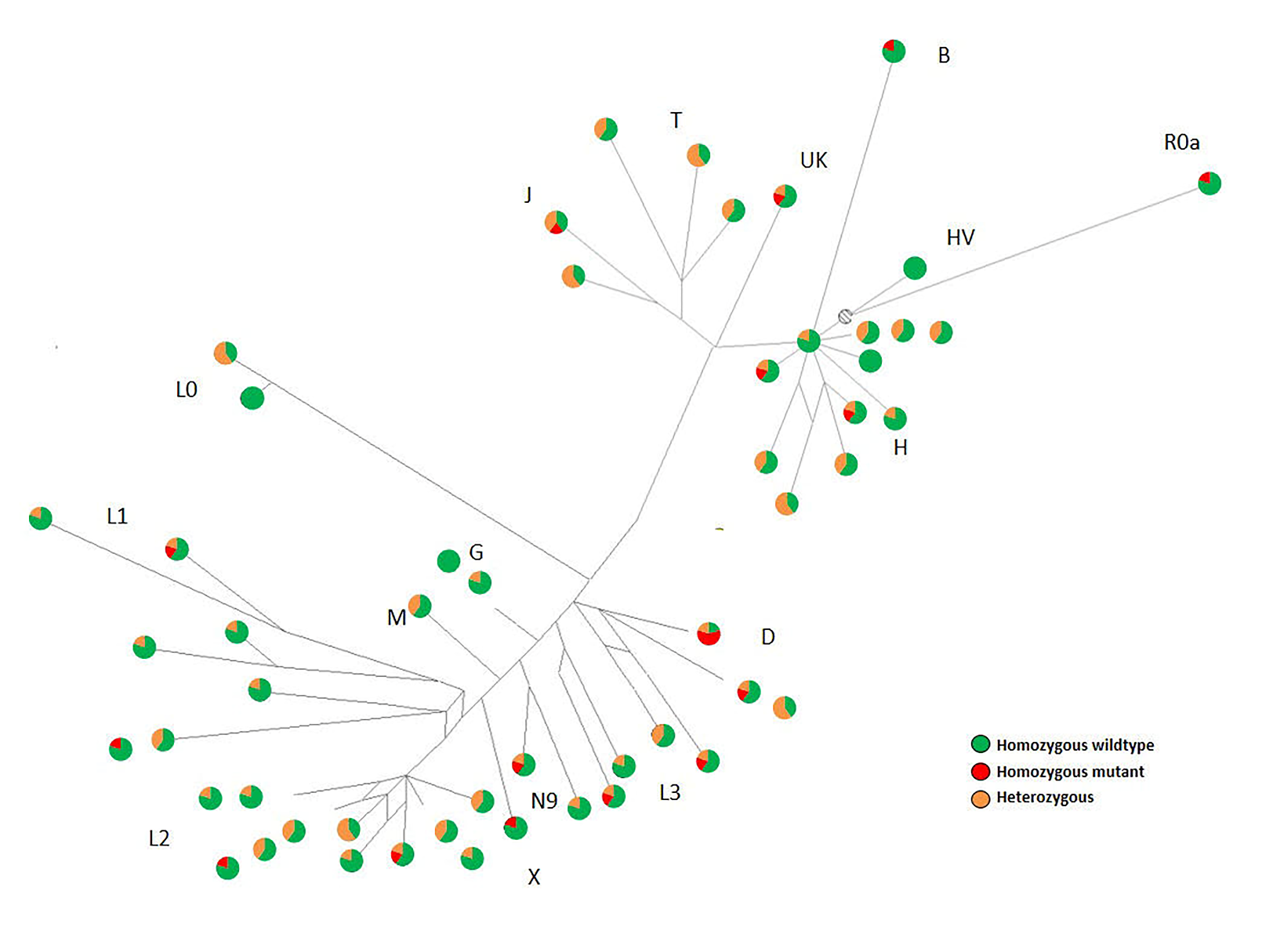
**
