# Supplementary material for: Comprehensive analysis of mitochondrial and nuclear DNA variations in patients affected by hemoglobinopathies: A pilot study
Source: PLoS One. 2020 Oct 22;15(10):e0240632. doi: 10.1371/journal.pone.0240632 (PMC7581000; doi:10.1371/journal.pone.0240632)
Supplement: S3 Fig — (A) Nuclear SNP frequency and Geographic distribution in β-thalassemia (β+/β+, β0/β0 and β+/β0) patients, (B) sickle cell/β-thalassemia (HbS/β+ or β0) patients, (C) sickle cell disease patients (HbS/HbS) (D) compound heterozygotes (HbS/HbC and HbO-Arab/HbC). Panel E-H: network plots obtained by including all mitochondrial and nuclear DNA variation, considered separately in each pathological group, and colored depending on their geographic origin. (DOCX) [file pone.0240632.s003.docx]

**S3 Fig. Nuclear SNP frequency**, **geographic distribution** **and** **network mtDNA,** **in the subgroups of patients.** (A) Nuclear SNP frequency and Geographic distribution in β-thalassemia (β^+^/β^+^, β^0^/β^0^ and β^+^/β^0^) patients, (B) sickle cell/β-thalassemia (HbS/β^+^ or β^0^) patients, (C) sickle cell disease patients (HbS/HbS) (D) compound heterozygotes (HbS/HbC and HbO-Arab/HbC). Panel E-H: network plots obtained by including all mitochondrial and nuclear DNA variation, considered separately in each pathological group, and colored depending on their geographic origin.

**
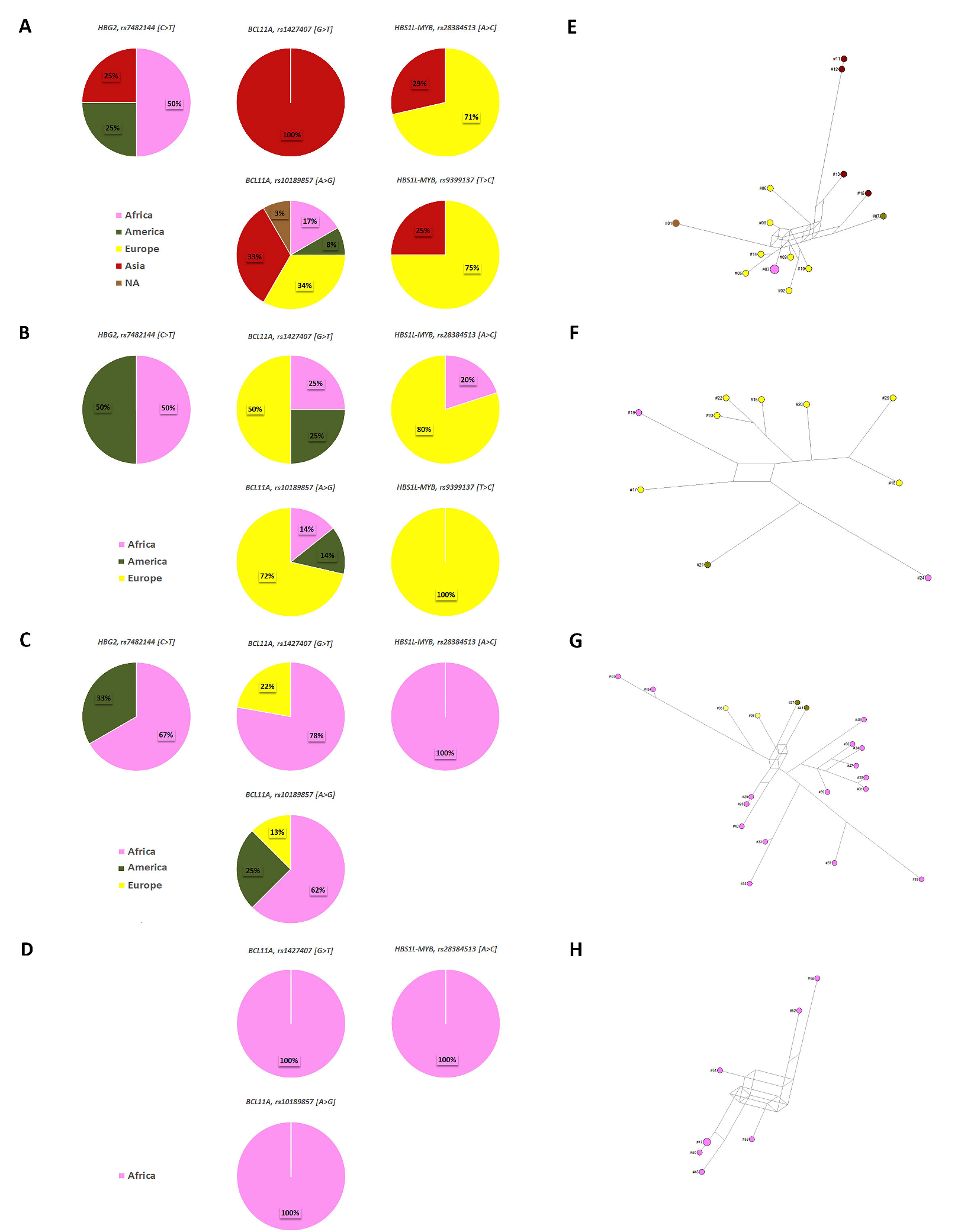
**
