# Supplementary material for: Comprehensive analysis of mitochondrial and nuclear DNA variations in patients affected by hemoglobinopathies: A pilot study
Source: PLoS One. 2020 Oct 22;15(10):e0240632. doi: 10.1371/journal.pone.0240632 (PMC7581000; doi:10.1371/journal.pone.0240632)

**S4 Fig. Network of mitochondrial variation and hemoglobinopathies**. Tree of the 53 mtDNA haplotypes observed among the patients in which different subgroups of hemoglobinopathies were highlighted.


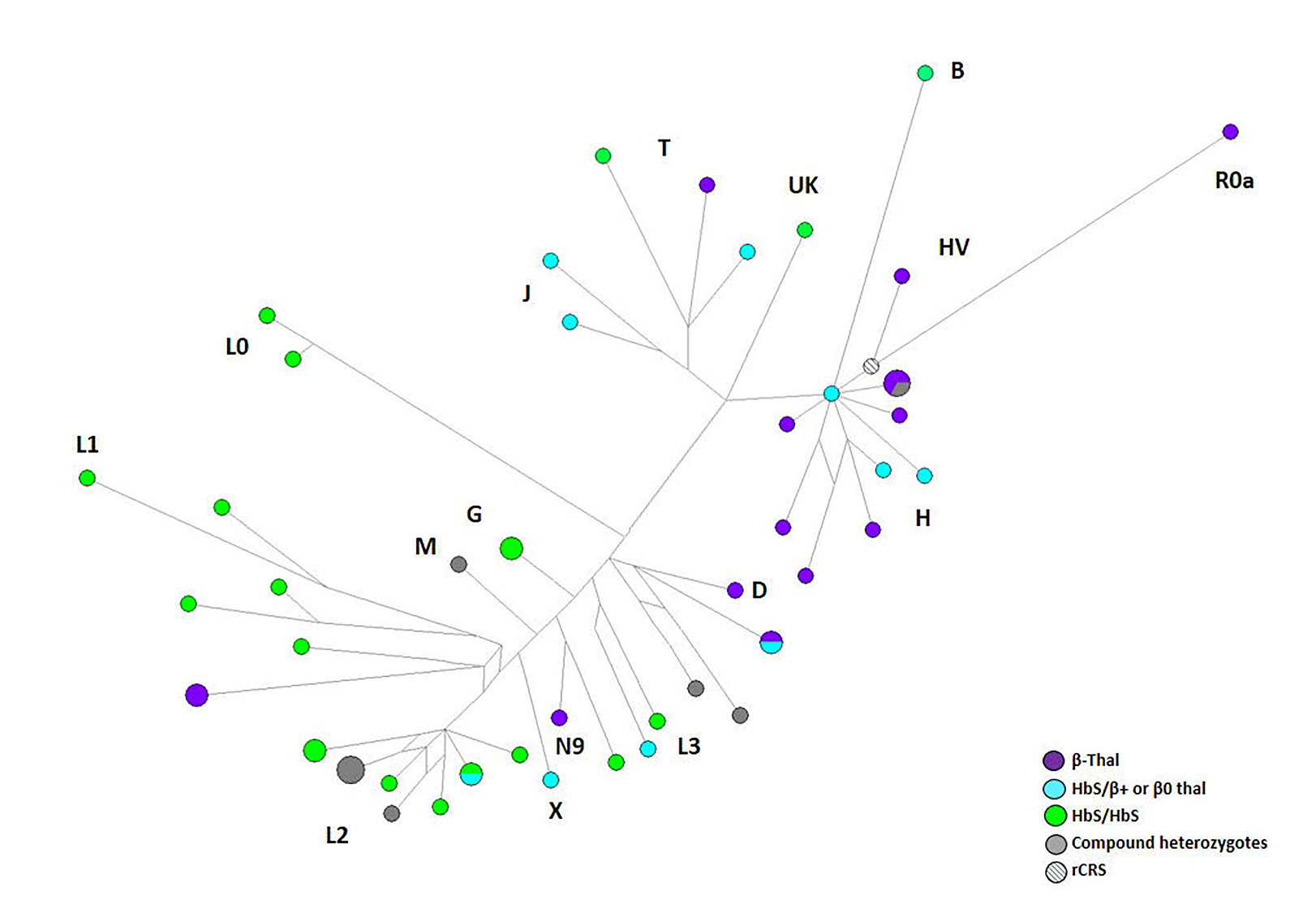

Supplement: S4 Fig — Tree of the 53 mtDNA haplotypes observed among the patients in which different subgroups of hemoglobinopathies were highlighted. (DOCX) [file pone.0240632.s004.docx]
