# Supplementary material for: Comprehensive analysis of mitochondrial and nuclear DNA variations in patients affected by hemoglobinopathies: A pilot study
Source: PLoS One. 2020 Oct 22;15(10):e0240632. doi: 10.1371/journal.pone.0240632 (PMC7581000; doi:10.1371/journal.pone.0240632)
Supplement: S1 Table — (DOCX) [file pone.0240632.s005.docx]

**S1 Table. Clinical data of β thalassemia patients.**

| **N°** | β **genotype** | **Transfusion frequency**  **(RBC/weeks)** | **HbF%** | **Age** |
| --- | --- | --- | --- | --- |
| 2 | codon 39(C>T)(β^0^) /codon 44(-C)(β^0^) | 2 RBC/2 weeks |  |  |
| 3 | - 29(A>G)(β^+^) / codon 8(-AA)(β^0^) | 2 RBC/2 weeks |  |  |
| 4 | - 29(A>G)(β^+^) / codon 8(-AA)(β^0^) | 1 RBC/2 weeks |  |  |
| 5 | codon 39(C>T)(β^0^) / IVS-I-6(T>C)(β^++^) | 1 RBC/3 weeks | 80 | 7 months |
| 6 | IVS-I-110(G>A)(β^+^) / IVS-I-110(G>A)(β^+^) | 1 RBC/3 weeks |  |  |
| 7 | codon 17(A>T)(β^0^) / - 29(A>G)(β^+^) | 1 RBC/3 weeks |  |  |
| 8 | IVS-I-5(G>C)/(β^+^) / IVS-I-6(T>C)(β^++^) | 1 RBC/3 weeks |  |  |
| 10 | codon 39(C>T)(β^0^) / IVS-I-6(T>C)(β^++^) | 1 RBC/2 weeks |  |  |
| 11 | codon 5 (-CT)(β^0^) / IVS-I-6(T>C)(β^++^) | 1 RBC/2 weeks |  |  |
| 12 | codon 5 (-CT)(β^0^) / IVS-I-6(T>C)(β^++^) | 1 RBC/3 weeks | 50.5 | 4 years |
| 13 | - 28(A>G)(β^+^) /cd17(A>T);AAG(Lys)>TAG(Stop codon)(β^0^) | 1 RBC/3 weeks |  |  |
| 14 | IVS-II-745 (C>G) (β^+^) / Hb Lepore Boston-Washington | 2 RBC/2 weeks |  |  |
