# Supplementary material for: Comprehensive analysis of mitochondrial and nuclear DNA variations in patients affected by hemoglobinopathies: A pilot study
Source: PLoS One. 2020 Oct 22;15(10):e0240632. doi: 10.1371/journal.pone.0240632 (PMC7581000; doi:10.1371/journal.pone.0240632)
Supplement: S2 Table — (DOCX) [file pone.0240632.s006.docx]

**S2 Table. Clinical data of Sickle Cell/Thalassemia patients.**

| **N°** | **β genotype** | **α genotype** | **Treatment** | **HbF%** | **Age** |
| --- | --- | --- | --- | --- | --- |
| 16 | Hb S / codon 39(C>T)(β^0^) | αα/αα | Phlebotomy / transfusion + HU |  |  |
| 17 | Hb S / codon 39(C>T)(β^0^) | ααα ^anti-3.7^/αα | EEX/HU |  |  |
| 18 | Hb S / codon 39(C>T)(β^0^) | αα/αα | RBC/HU |  |  |
| 19 | Hb S / -29(A>G)(β^+^) | αα/αα | - | 2.9 | 6 years |
| 20 | Hb S / IVS-I-6(T>C)(β^++^) | αα/αα | - | 0.8 | 46 years |
| 21 | Hb S / -29(A>G)(β^+^) | αα/αα | No info available |  |  |
| 22 | Hb S / IVS-I-110(G>A)(β^+) | αα/αα | EEX/HU |  |  |
| 23 | Hb S / IVS-I-110(G>A)(β^+^) | αα/αα | RBC/HU | 11.6 | 18 months |
| 24 | Hb S / -29(A>G)(β^+^) | αα/αα | Sporadic RBC | 5.8 | 19 years |
| 25 | Hb S / codon 39(C>T)(β^0^) | ααα ^anti-3.7^/αα | HU | 25.5 | 24 years |
