# Supplementary material for: Comprehensive analysis of mitochondrial and nuclear DNA variations in patients affected by hemoglobinopathies: A pilot study
Source: PLoS One. 2020 Oct 22;15(10):e0240632. doi: 10.1371/journal.pone.0240632 (PMC7581000; doi:10.1371/journal.pone.0240632)
Supplement: S3 Table — (DOCX) [file pone.0240632.s007.docx]

**S3 Table. Clinical data of Sickle Cell disease patients.**

| **N°** | **α genotype** | **Treatment** | **HbF%** | **Age** |
| --- | --- | --- | --- | --- |
| 26 | αα/αα | HU | 3.4 | 7 years |
| 27 | αα/αα | ND | 4.7 | 32 years |
| 28 | - α ^3.7^/αα | HU | 18.7 | 4 years |
| 29 | αα/αα | ND | 34.6 | 5 months |
| 30 | αα/αα | HU |  |  |
| 31 | αα/αα | RBC prophilaxys | 10.8 | 2 years |
| 32 | αα/αα | HU | 12.4 | 5 years |
| 33 | - α ^3.7^/αα | Sporadic RBC + HU | 20 | 2 years |
| 34 | αα/αα | Sporadic RBC + HU | 30 | 5 months |
| 35 | - α ^3.7^/αα | EEX | 4 | 49 years |
| 36 | αα/αα | Lost FU | 10.5 | 2 years |
| 37 | αα/αα | Sporadic RBC | 45 | 1 year |
| 38 | - α ^3.7^/αα | HU | 3 | 14 years |
| 39 | αα/αα | ND |  |  |
| 40 | - α ^3.7^/αα | None | 16.3 | 12 years |
| 41 | αα/αα | Sporadic RBC + HU |  |  |
| 42 | α-^3.7^/α-^3.7^ | Sporadic RBC | 1.6 | 60 years |
| 43 | αα/αα | Lost FU | 24.5 | 9 months |
| 44 | αα/αα | Lost FU |  |  |
| 45 | αα/αα | Sporadic RBC | 11.2 | 14 months |
