# Supplementary material for: Comprehensive analysis of mitochondrial and nuclear DNA variations in patients affected by hemoglobinopathies: A pilot study
Source: PLoS One. 2020 Oct 22;15(10):e0240632. doi: 10.1371/journal.pone.0240632 (PMC7581000; doi:10.1371/journal.pone.0240632)
Supplement: S4 Table — (DOCX) [file pone.0240632.s008.docx]

**S4 Table. Clinical data of Compound Heterozygotes patient.**

| **N°** | **β genotype** | **α genotype** | **Treatment** | **HbF%** | **Age** |
| --- | --- | --- | --- | --- | --- |
| 46 | HbS / Hb C | -α^3.7^/αα | Lost FU | 1.3 | 38 years |
| 47 | HbS / Hb C | αα/αα | None | 12.8 | 23 months |
| 48 | HbS / Hb C | αα/αα | Lost FU | 24.1 | 1 year |
| 49 | HbS / Hb C | -α^3.7^/αα | None | 1.4 | 14 years |
| 50 | HbS / Hb C | αα/αα | None | 3.4 | 16 years |
| 51 | HbS / Hb C | αα/αα | None | 4.4 | 24 years |
| 52 | Hb C / Hb O-Arab | αα/αα | None | 0.7 | 59 years |
| 53 | HbS / Hb C | αα/αα | Lost FU | 3 | 17 years |
