# Supplementary material for: Comprehensive analysis of mitochondrial and nuclear DNA variations in patients affected by hemoglobinopathies: A pilot study
Source: PLoS One. 2020 Oct 22;15(10):e0240632. doi: 10.1371/journal.pone.0240632 (PMC7581000; doi:10.1371/journal.pone.0240632)
Supplement: S5 Table — (DOCX) [file pone.0240632.s009.docx]

**S5 Table. Molecular characteristics of the genetic modulators of clinical phenotypes in homozygotes and compound heterozygotes for β gene defect.**

| **Genetic Modifiers** | **Patients** | **β-Thal** | **HbS/β^+^ or β^0^ thal** | **HbS/HbS** | **Compound heterozygotes** |
| --- | --- | --- | --- | --- | --- |
| Patients, *n* (%) | 53 (100.0) | 15 | 10 | 20 | 8 |
| α-globin genes, *n* (%)  αα/ αα  - α ^3.7^/αα  - α ^3.7^/- α^3.7^  ααα ^anti3.7^ | 43 (81)  7 (13)  1 (2)  2 (4) | 15  0  0  0 | 8  0  0  2 | 14  5  1  0 | 6  2  0  0 |
| *HBG2*, rs7482144 (T), *n* (%) | 9 (17) | 4 | 2 | 3 | 0 |
| *BCL11A*  rs10189857, (G), *n* (%)  rs1427407, (T), *n* (%) | 33 (62)  19 (36) | 12  1 | 7  4 | 8  9 | 6  5 |
| *HBS1L-MYB intergenic region*  rs9399137, (C), *n* (%)  rs28384513, (C), *n* (%) | 6 (11)  21 (40) | 4  7 | 2  5 | 0  7 | 0  2 |
