# Supplementary material for: Comprehensive analysis of mitochondrial and nuclear DNA variations in patients affected by hemoglobinopathies: A pilot study
Source: PLoS One. 2020 Oct 22;15(10):e0240632. doi: 10.1371/journal.pone.0240632 (PMC7581000; doi:10.1371/journal.pone.0240632)
Supplement: S1 Text — (DOCX) [file pone.0240632.s010.docx]

**Supporting information**

**S1 Text**

**β-thalassemia patients (β^+^/β^+^, β^0^/β^0^ and β^+^/β^0^)**

The β-globin genotyping confirmed the diagnosis of homozygous β-thalassemia in 4/15 cases while 11/15 were a double heterozygous β-thalassemia, 1/15 was HbE homozygous (patient 15) and in 1/15 no clinical information was available although genotype addresses to a β-thalassemia major (patient 1). Twelve (80%) were transfusion-dependent (TD); frequency and genotype were reported in S1 Table 1. One TD patient (patient 11) had a successful hematopoietic stem cell transplantation from his HLA identical sibling when he was 4 years old. 15/15 patients showed a normal α genotype (αα/αα).

**Sickle cell/β-thalassemia patients (HbS/β^+^ or β^0^)**

For one out of ten patients no clinical information was available (patient 21). One patient developed a Burkitt Lymphoma at 34 years of age and obtained complete remission after chemotherapy (follow-up 6 months) (patient 17). Only two patients (patients 19, 20) were out of treatment (due to an apparently mild phenotype). One patient developed one painful crisis/year between 2014 and 2016 without RBC requirement (patient 19); one died after an acute chest syndrome at 49 years old (patient 20). One had transfusion support only during a sepsis and made hip prosthesis following osteonecrosis (patient 24). Three patients (18, 23 and 25) responded well to hydroxyurea (HU), reducing/suspending the transfusions (patients 18, 23 and 25) (see S2 Table). One (patient 18) underwent splenectomy at eleven years old. Patients 16 and 22 are on HU and red blood cells exchange (patients 16 manual and 22 automatic) due to recurrence of painful crisis (S2 Table).

**Sickle cell disease patients (HbS/HbS)**

Three patients died (27, 29 and 39); for 27 and 29 we have no clinical data before death; for 29, who was patient 28s brother, we know that he required only one RBC before death, due to Varicella -zoster virus infection infection. We lost at follow-up patients 36, 43 and 44; patient 36 starts HU in 2015 after previous RBC prophylaxis due to recurrent chest-crisis, with only a reduction of frequency. Five patients (26, 28, 30, 32 and 38) suspended RBC prophylaxis after the beginning of HU treatment, with marked reduction of pain crises. Patients 31, who was patient 30’s brother, was intolerant to HU and entered RBC prophylaxis for high blood flow velocity at TCD (Transcranial Doppler Ultrasound).

Patients 33, 34 and 41 suspended RBC prophylaxis after starting HU treatment. Patient 33 needed sporadic transfusions for recurrent episodes of splenic infarcts, while patient 34 had frequent acute chest syndromes and underwent HSCT on June 2019 from an identical sibling donor. Patient 41 still requires RBC for anemia, without significant recurrence of painful crises. Patient 35 underwent erythrocyte exchange (EEX) because of recurrent painful crises despite HU treatment. Patients 37, 42 and 45 received sporadic transfusions for anemia without indication to RBC prophylaxis (normal TCD, low recurrence of painful crisis, no acute chest syndrome). Patient 40 needed no treatment because so far he only underwent one painful crisis (the patients is 14 years old). The α-globin genotyping was reported in S3 Table.

**Compound heterozygotes patients (HbS/HbC and HbC/O-Arab)**

Two out of eight patients (patients 46 and 51) were subjected to EEX. Patient 46 is now lost to FU (follow-up), while 51 suspended EEX and did not experience painful crisis (the patient refused HU treatment because of her desire to became pregnant). Patients 47-50, 52 and 53 did not underwent any treatment. In particular, patient 53 made only one RBC during an episode of painful crisis and was lost at FU since 2017. Patient 48 was lost to FU from 2016, before this date she did not develop any clinical problem related to hemoglobinopathy. The β and α-globin genotyping was reported in S4 Table.
