# Supplementary material for: Comprehensive analysis of mitochondrial and nuclear DNA variations in patients affected by hemoglobinopathies: A pilot study
Source: PLoS One. 2020 Oct 22;15(10):e0240632. doi: 10.1371/journal.pone.0240632 (PMC7581000; doi:10.1371/journal.pone.0240632)
Supplement: S2 Text — (DOCX) [file pone.0240632.s011.docx]

**S2 Text**

In order to better characterize and stratify our patients we analysed the nuclear SNPs considering four subgroups: β-thalassemia, sickle cell/β-thalassemia, sickle cell disease (HbS/HbS) and compound heterozygotes. For details see Table 2, S2A-D Figure and S5 Table.

**β-thalassemia subgroup**

In 13 out of 15 analyzed patients at least one alteration was found and only 2/15 (patients 5 and 14) were negative for all investigated SNPs (Table 2, S5 Table). Moreover, patients 3 and 4 (brothers) showed the same SNP-genotype, while patients 11-12 (brothers) showed different genotypes.

*BCL11A*, rs1427407 [G>T] was the less polymorphic alteration, with just one mutated patient from Asia (patient 11). Differently, *BCL11A* rs10189857 [A>G] and *HBS1L-MYB*, rs28384513 [A>C] were the most polymorphic variations. In particular, rs10189857 G allele, was detected in 12/15 patients (six homozygotes and six heterozygotes). These patients came from four continent: four Europe, two Africa , four Asia and one from South America.

*HBS1L,* rs28384513 A>C, was detected in 7/15 patients (one homozygote and six heterozygotes). Five patients came from Europe and two from Asia, while American and African patients carrying rs28384513 A>C were not found. Similarly, *HBS1L-MYB* rs9399137 [T>C], was detected only in 4/15 patients, three European and one Asian.

Finally, *HBG2* rs7482144 [C>T] was detected in 4/15 patients: two Africans, one South American and one Asian. No European patients carrying this SNP were identified.

**Sickle cell/β-thalassemia subgroup (HbS/β^+^ or β^0^)**

In all patient at least one gene variation was detected (Table 2, S5 Table). The less polymorphic variants were HBG2, rs7482144 [C>T] and *HBS1L-MYB,* rs9399137 [T>C], both detected in only two patients. *HBG2*, rs7482144 [C>T] was detected in one African patient and in one American patient (patients 21 and 24). It was not found in European patients. Conversely *HBS1L-MYB*, rs9399137 [T>C] was detected only in European patients (patients 18 and 25)

*BCL11A* rs10189857 [A>G] and *HBS1L* rs28384513 [A>C] were the most polymorphic variations. In particular, *BCL11A* rs10189857 [A>G] was identified in 7/10 patients (2 homozygotes and 5 heterozygotes). Five of these patients were Europeans, one African and one came from America. *HBS1L* rs28384513 [A>C] was present in 5/11 patients (heterozygotes patients: 16, 18-20, 25): four Europeans and one African patient. It was not found in American patients.

*BCL11A* rs1427407 [G>T] was detected in 4/10 patients: two Europeans, one African and one American.

**Sickle cell disease subgroup (HbS/HbS)**

In 18 out of 20 analysed patients at least one genomic variant was detected while in two patients (patients 29 and 45) no genetic variants were identified.

Differently to the thalassemia and microdrepanocytosis subgroups, no patients with *HBS1L-MYB* rs9399137 [T>C] were identified. *HBG2* rs7482144 [C>T] was detected only in 3/20 patients (patients 39-41): two African and one American patient while it was not detected in European patients.

Otherwise, *BCL11A* rs1427407 [G>T] was detected in 9/20 patients (one homozygotes and eight heterozygotes). Seven patients came from Africa and two from Europe while it was not identified in American patients.

*BCL11A* rs10189857 [A>G], was identified in 8/20 patients: five Africans, two Americans and one European. Three patients were homozygous and five were heterozygous.

Moreover, *HBS1L-MYB* rs28384513 [A>C], was identified in 7/20 patients come from Africa.

Two couples of brothers were analysed (patients 28, 29 and 30, 31). Brothers 28 and 29 showed different genotypes: rs1427407 [G>T] was present in patient 28 while no alterations were identified in patient 29. Similarly, *BCL11A* rs1427407 [G>T] was present in patient 30 while rs10189857 [A>G] was detected in his brother (patient 31).

**Compound heterozygotes subgroup (HbS/HbC and HbC/O-Arab)**

In all patients at least one genetic variation was identified. *BCL11A* rs1427407 [G>T] and rs10189857 [A>G] were the most polymorphic variants, detected respectively in 5/8 patients (one homozygote: patient 50, and four heterozygotes patients: 46-47, 49 and 51) and in 6/8 patients (one Homozygotes patient 48, and five heterozygotes patients: 47, 49 and 51-53). *HBS1L-MYB* rs28384513 [A>C] was detected, in heterozygosity, in two patients (48 and 52). All patients were Africans. No patients carrying *HBS1L-MYB* rs9399137 [T>C] and *HBG2* rs7482144 [C>T] were found.
